# Deep Anomaly Detection for CNC Machine Cutting Tool Using Spindle Current Signals

**DOI:** 10.3390/s20174896

**Published:** 2020-08-29

**Authors:** Guang Li, Yan Fu, Duanbing Chen, Lulu Shi, Junlin Zhou

**Affiliations:** 1 Big Data Research Center, University of Electronic Science and Technology of China, Chengdu 611731, China; leeguang923@gmail.com (G.L.); fuyan@uestc.edu.cn (Y.F.); dbchen@uestc.edu.cn (D.C.); 201721060404@std.uestc.edu.cn (L.S.); 2 Union Big Data, Chengdu 610000, China

**Keywords:** tool breakage, deep learning, anomaly detection, spindle current

## Abstract

In recent years, industrial production has become more and more automated. Machine cutting tool as an important part of industrial production have a large impact on the production efficiency and costs of products. In a real manufacturing process, tool breakage often occurs in an instant without warning, which results a extremely unbalanced ratio of the tool breakage samples to the normal ones. In this case, the traditional supervised learning model can not fit the sample of tool breakage well, which results to inaccurate prediction of tool breakage. In this paper, we use the high precision Hall sensor to collect spindle current data of computer numerical control (CNC). Combining the anomaly detection and deep learning methods, we propose a simple and novel method called CNN-AD to solve the class-imbalance problem in tool breakage prediction. Compared with other prediction algorithms, the proposed method can converge faster and has better accuracy.

## 1. Introduction

Modern industry is increasingly demanding automation, and automatic management of computer numerical control (CNC) has great significance for the industry [1,2]. In a real manufacturing process, breakage of machine tools usually happens without any indication, which brings to the high defect rate and production cost. The task of tool breakage monitoring aims at accurately detecting the tool wear state in real-time, which often consists of four parts—sensor data acquisition, data processing, feature extraction and modeling. The problem of data cost and class-imbalance is the difficulty of tool wear monitoring. In this work, we propose a new framework to explore a tool monitoring system who emphasizes on the actual scene. The hall sensor is used for collecting current data of machine spindle in our system, which can achieve low cost and high real-time data acquisition. Then, We use frequency domain technology to filter and denoise the signal, and time domain technology to feature extraction. Finally, we propose a novel method called CNN-AD, which is trained by only normal machining signals, to solve the class-imbalance problem and has stronger generalization ability.

As input to the tool breakage monitoring system, a suitable physical signal is crucial for understanding the process of tool wear, the common used physical monitoring signals include acoustic emission, vibration force, spindle current, cutting force, cutting temperature and so on [3,4,5]. In traditional tool wear monitoring, Li proposed a method to detect tool flute breakage by monitoring the features which are generated through improved time-domain averaging (TDA) decomposes the feed-motor current signals [6]. Nie detected tool wear by using db8 to decompose the Acoustic Emission (AE) signals and extracting the feature vector of frequency band [3]. Akbari proposed a method that monitoring the tools wear by quantizing the harmonic distortion of spindle current and characteristic analysis in time and frequency domain [7]. In these studies, features are selected manually by signal analysis to observe tool wear changes, but the features used are relatively single and do not have the learning ability, so they are often poor in judging complex and changeable signals. With advances in sensor and computer technology, more and more sensor signals and statistical learning methods are incorporated into tool breakage monitoring systems. Lian proposed a method that extracted the features by singular value decomposition (SVD) and classified tool breakage through Linear Discriminant analysis (LDA) based on spindle current to monitor the tool breakage [8]. Lin proposed a method of least-squares support vector machine (LS-SVM) which is trained by the feature extracted with Sym6 wavelet transformation to decompose the feedback signals [9]. Xu proposed a method of Empirical Mode Decomposition (EMD) and support vector machine (SVM) based on AE signal, AE signals is decomposed by EMD to achieve Intrinsic Mode Functions (IMFs), then feature vectors are calculated by IMFs and SVM is trained by this feature vectors [10]. The training of SVM often needs to reduce the dimension in a wide range, such as principal component analysis (PCA), which will lead to the omission of certain signal features. Neural networks are also corporated into tool monitoring. Li proposed a method of a fuzzy network to defect tool wear based on current [11]. Corne analyzed the feasibility of prediction tools based on current and the performance of different neural networks [12,13].

The existing studies improve the accuracy and diversity of tool wear prediction, but there are still some problems—(1) The cost of model training is expensive, supervised learning requires a large number of training sets, but tool breakage often means that defective goods will be produced, which leads to high cost. (2) Due to the class-imbalance, the high accuracy of the model is often reflected in the good performance of normal wear prediction, but in fact, the prediction accuracy of tool breakage is poor. (3) The generalization ability of these methods is not enough. It is likely to make a prediction error when an untrained breakage occurs, and this is common in practical processing because the form of tool chipping is often random. Anomaly detection refers to the problem of finding patterns in data that do not conform to expected behavior [14]. Getting a labeled set of anomalous data instances which cover all possibilities of anomalous behavior is more difficult than getting labels for normal behavior. Moreover, the anomalous behavior is often dynamic in nature. Tool breakage detection is a typical anomaly detection problem, tool breakage samples are only a little, while the normal machining samples are abundant.

In summary, our key contributions are—(1) A tool breakage monitoring system is proposed for low-cost, high real-time and accurate detection. (2) CNN-AD has strong generalization ability when there are differences between tool breakage and normal machining signals. (3) Combining the anomaly detection and deep learning methods, CNN-AD solves the class-imbalance problem because it only uses normal machining signals for training. we perform extensive experiments on the actual processing dataset, compared with other prediction algorithms, it is found that CNN-AD can converge faster and have better performance.

## 2. Materials and Methods

We use the spindle current data as experimental data. In this section, we discuss the data environment and the main method used in the experiment.

### 2.1. Data Environment

The experimental data is collected in the real production process of a well-known manufacturer. We install the Hall sensor on the spindle control wire of the machine tool, and the current signal is sliced and stored in the memory after the data acquisition card performs analog-to-digital conversion. Experimental environment information of processing is shown in Table 1.

Different experimental environments or even subtle changes in machine parameters will affect the collected data. The most important parameters of this machine we must notice are as follows:

- Methods of processing, like Turning, Milling, Grinding, Drilling, Wire Cutting. Different processing methods will mean the extent and type of wear on the tool are different.
- Rotating speed, different processing requires different rotating speed. When the rotating speed is high, it will lead to high temperature due to high-speed friction during tool processing, which will change the structure and composition of the tool material, and it is also more likely to cause tool wear.
- Feed rate, which represents the degree of tool movement relative to the workpiece in the direction of feed motion. The size of the feed rate not only determines the machining efficiency of the machine tool, but also has an important impact on the surface quality of the workpiece. If the feed rate changes sharply, it will seriously affect the service life of the tool and the CNC machine.

Considering the real-time requirements in actual processing, we use Hall Current Sensor and NI PCIe data acquisition card to acquire the spindle current of the machine with the sampling frequency of 20,000 points per second in this work.

### 2.2. Definition of Abnormal and Data Category

In our work, an important statement needs to be declared. We regard tool breakage as an abnormal case, and general machining as a normal case. The abnormal division of the sample is usually based on the experience of the worker, who judges according to the machining noise and the quality of the workpiece. The processing noise of machine tools will go through the experience from slight to harsh. When the tool breakage occurs, the noise is harsh and the vibration of the machine tool will increase. In addition, when the machining specification of the workpiece is not up to standard and the processing surface is rough, we also think the breakage has occurred.

To prevent confusion, categories of data also need to be defined. In the experiments, the processing current data with a duration of 6 min is regarded as a sample, which is divided into two categories: positive corresponding to normal state and negative to abnormal state. The label of sample depends on the tool status at the 7th minute. For example, at the 47th minute, the tool breakage occurred, then, the label of samples from 41 to 46 min was divided to negative. In addition, the samples after 47 min were also classified as negative.

### 2.3. Feature Analysis Methods

#### 2.3.1. Time Domain Feature Analysis

Time domain signal refers to the measurement sequence with time as variable, which is completely localized in the time domain. it can accurately and intuitively reflect the change of the tool at each moment during the cutting process. we can extract relatively stable and reliable monitoring indicators through time-domain analysis. The common time domain analysis methods are as follows:

- Mean, which represents the central trend of the tool condition monitoring signal, is a first-order statistical feature. With the increase of tool wear, the mean value of sensor signal will change in different degrees. Defined as:
  (1) $$\bar{x}=\frac{1}{N}\sum_{i=1}^{N}\left|x_{i}\right|.$$
- Variance, describes the dynamic fluctuation degree of the signal. Defined as:
  (2) $$\sigma^{2}=\frac{1}{N}\sum_{i=1}^{N}{\left(x_{i}-\bar{x}\right)}^{2}.$$
- Peak-to-peak value, is the difference between the peak and trough of a signal, used to describe the degree of fluctuation of the signal. Defined as:
  (3) $$X=max\left(x_{i}\right)-min\left(x_{i}\right).$$
- Kurtosis describes the sharpness of the peak of a frequency-distribution curve and the flatness of the data distribution. Kurtosis can often get a good result in anomaly detection problems. A high Kurtosis means that the increase in variance is caused by an extreme difference in which the low frequency is either greater or less than the average, as:
  (4) $$K=\frac{\frac{1}{n}\sum_{i=1}^{N}{\left(x_{i}-\bar{x}\right)}^{4}}{{\left(\frac{1}{n}\sum_{i=1}^{N}{\left(x_{i}-\bar{x}\right)}^{2}\right)}^{2}}.$$
- Skewness describes the asymmetry of the probability distribution of real random variables. A skewness of zero means that data are evenly distributed on both sides of the average, negative skewness means that most of the data are on the right side of the average. Conversely, positive values of skewness indicated that most of the data are on the left side of the average, as:
  (5) $$S=\frac{\frac{1}{n}\sum_{i=1}^{N}{\left(x_{i}-\bar{x}\right)}^{3}}{{\left(\frac{1}{n}\sum_{i=1}^{N}{\left(x_{i}-\bar{x}\right)}^{2}\right)}^{\frac{3}{2}}}.$$

In the experiment, we performed the above-mentioned time domain analysis with a time span of 1 s and 1 min to observe the changes in tool wear in the time domain. Then, the features that are sensitive to tool wear are selected as the input of the final model.

#### 2.3.2. Frequency Domain Feature Analysis

Generally, the signals measured by the sensors are time domain signals, but the tool breakage often causes the change of signal frequency distribution. Therefore, it is necessary to analyze the signal in frequency domain. Frequency domain analysis can obtain the frequency structure, and the relationship between amplitude, phase energy and frequency. The typical methods as follows:

- Frequency spectrum:
  (6) $$X\left(\omega \right)=\int_{-\infty }^{+\infty }x\left(t\right)e^{-j\omega t}dt.$$
- Power spectrum:
  (7) $$S_{x}\left(\omega \right)=\left|F{\left(\omega \right)}^{2}\right|=\int_{-\infty }^{+\infty }R_{x}\left(\tau \right)e^{-j\omega t}dt,$$

where $R_{x}\left(\tau \right)$ is an autocorrelation function.

#### 2.3.3. Wavelet Packet Analysis

Many studies extract characteristics through wavelet packets [15,16]. Frequency domain analysis describes the spectral characteristics of the signal, but it can not indicate both the time and frequency local information of the signal. In order to solve this problem, the wavelet packet transform is used to get the local characteristics of the time-frequency domain. Given orthogonal scaling function j(t) and wavelet function f(t), as:

(8) $$\left\{\begin{array}{c} \varphi \left(t\right)=\sqrt{2}\sum_{k}h_{0k}\varphi \left(2t-k\right)\\ \phi \left(t\right)=\sqrt{2}\sum_{k}h_{0k}\phi \left(2t-k\right), \end{array}\right.$$

where $h_{0k},h_{1k}$ are the filter coefficients. In order to further extend the two scale equation, the following recurrent relation is defined.

(9) $$\left\{\begin{array}{c} w_{2n}\left(t\right)=\sqrt{2}\sum_{k\in Z}h_{0k}w_{n}\varphi \left(2t-k\right)\\ w_{2n+1}\left(t\right)=\sqrt{2}\sum_{k\in Z}h_{1k}w_{n}\varphi \left(2t-k\right), \end{array}\right.$$

where $w_{0}\left(t\right)=\phi \left(t\right)$ is a scaling coefficient and $w_{1}\left(t\right)=\varphi \left(t\right)$ is a wavelet coefficient. The set of functions $w_{n}{\left(t\right)}_{n\in Z}$ are wavelet packets. In order to discuss the space of wavelet packets, the scale subspace $V_{j}$ and the wavelet subspace $W_{j}$ are unified to be characterized by a new subspace $U_{j}^{n}$,and the following symbols are introduced as:

(10) $$\left\{\begin{array}{c} U_{j}^{0}=V_{j},j\in Z\\ U_{j}^{1}=W_{j},j\in Z. \end{array}\right.$$

According to the wavelet multiresolution analysis, there are:

(11) $$V_{j+1}=V_{j}⨁W_{j},j\in Z.$$

The introduced wavelet packet symbols are expressed as:

(12) $$U_{j}^{0}=U_{j+1}^{0}⨁U_{j+1}^{1},j\in Z.$$

Then extension to the wavelet packet:

(13) $$U_{j}^{n}=U_{j+1}^{2n}⨁U_{j+1}^{2n+1},j\in Z,n\in Z^{+},$$

where $U_{j+1}^{2n}$ and $U_{j+1}^{2n+1}$ are the subspaces of $U_{j}^{n}$, $U_{j+1}^{2n}$, corresponds to $w_{2n}$, and $U_{j+1}^{2n+1}$ corresponds to $w_{2n+1}$. The decomposition space of multiresolution analysis is $L^{2}\left(R\right)={⨁}_{j\in Z}W_{j}$. In order to make it easier to compare, we use $W_{j}^{n}$ to represent $U_{j}^{n}$, and the general expression of wavelet packet decomposition is as follows:

(14) $$\left\{\begin{array}{c} w_{j}=U_{j+1}^{2}⨁U_{j+1}^{3}\\ w_{j}=U_{j+2}^{4}⨁U_{j+2}^{5}⨁U_{j+2}^{6}⨁U_{j+2}^{7}\\ \vdots \\ w_{j}=U_{j+k}^{2^{k}}⨁U_{j+k}^{2^{k}+1}⨁U_{j+k}^{2^{k+1}}⨁U_{j+k}^{2^{k+1}-1}. \end{array}\right.$$

**Figure 1:**
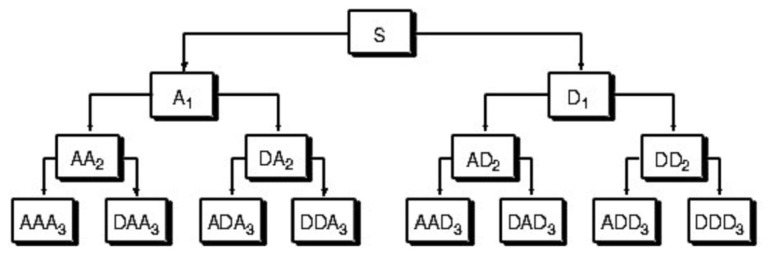
Three-layer wavelet packet decomposition.

The decomposition process of wavelet packets is shown in Figure 1:

The original frequency band is divided into two layers in each layer, and the original frequency band is divided into $2^{k}$ subbands for the *k* layer wavelet packet, then, the subdivision frequent band is realized, and the resolution of the frequency domain is improved. Before the wavelet packet feature is extracted, the wavelet packet decomposition level should be determined first. After the signal is decomposed by wavelet packet, the band resolution of each wavelet packet decomposition is defined as follows:

(15) $$f=(\frac{FS}{2}/2^{n}),$$

where FS represents the signal sampling frequency, *n* is the number of wavelet decomposition layers. Not each frequency-band energy can reflect the change of tool breakage. Therefore, if the number of decomposition layers is too large, the number of interference features also increases. But if the decomposition level is little, the resolution of the frequency band is very small, and it may not be able to accurately extract the frequency band characteristic values that reflect the status of a tool. In this paper, through comparing the characteristics of the spectrum distribution of signals in different situations, we decide the frequency band resolution, and the number of decomposition layers which is deduced by Equation (15).

### 2.4. Wear Prediction Methods

#### 2.4.1. Convolutional Neural Network

Convolutional Neural Network (CNN) is one of the most representative network structures in deep learning and has achieved great success in supervised learning [17,18,19,20,21]. In addition to strong fitting and learning ability, CNN has some other advantages compared with traditional neural networks, as follows:

- The CNN extracts the local features of the signals by convolution kernels, which is composed of some parameters. The convolution kernels slides on input data with set step size to calculate weighted average, such as:
  (16) X=W·X,
  where *W* is the convolution kernel matrix and *X* is the equal-sized input data block corresponding to the convolution kernels. The parameters in the convolution kernel matrix will increasingly favor the inputs or locations that have a significant impact on the outcome as the network is optimized. For example, when we observe a person, we focus on his nose, hair, eyes, and so forth, and these positions are important features of him.
- Weight sharing. For the different inputs, the same convolution kernel matrix is used to extract features, which is like using the same pair of eyes for observing different images. In this way, CNN can save a lot of parameters compared to traditional neural networks. At the same time, this weight sharing structure reduces the complexity of the network and prevents over-fitting to a certain extent.

#### 2.4.2. Convolution Neural Network Framework In Experiments

The performance of CNN will be affected by some super parameters, which are shown in Table 2.

- Depth of network, the neural network is composed of many parameters. If the network is deep and has many parameters, the network will have greater learning ability and capacity, but it may also lead to over-fitting, and vice versa. With this in mind and after testing, we chose a compromise depth.
- Learning rate is one of the most important hyperparameters of CNN. When we choose a large learning rate, the loss function may miss the best point and reach the other direction of the gradient. If the learning rate is too small, the network will move slowly, and it may be lost in the local optimum. So, a suitable learning rate can help us to reach the best point quickly. After repeated adjustments, we finally set the learning rate with 0.0001.

**Figure 2:**
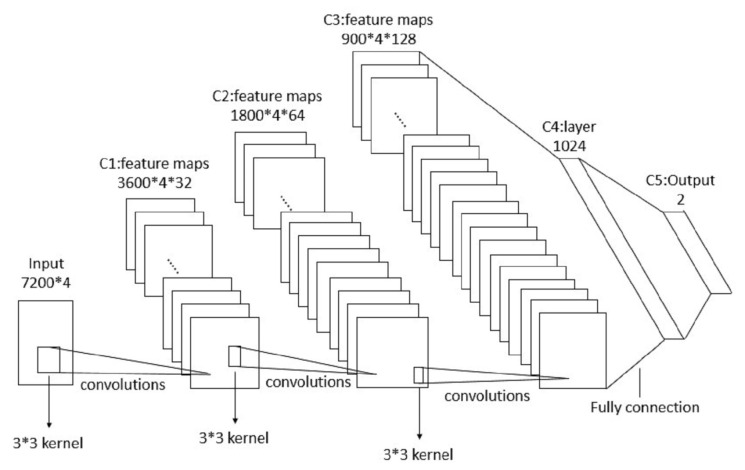
The architecture of convolutional neural network (CNN).

The architecture of CNN is shown in Figure 2.

The convolutional neural network we built has an input layer, three hidden layers, and a fully connected layer. Finally, we get the output of prediction results by the softmax layer. The data volume we used is contained by the whole life cycle of five tools spindle current signals, totals of 404 samples. The data of size 4 × 7200 × 1 into the network, we choose zero-padding if the data size does not reach 7200. Deserved to be mentioned, input data is randomly selected in the train step, but in the test step, it is extracted by the time sequence of tool processing.

All the strides of the pooling layer we used are [1 × 2] and size of kernels is also [1 × 2]. A pooling layer is connected after each convolutional layer and a ReLu layer is connected after each normalization layer. Considering that small convolution can extract more detailed information, in the experiment, all of our convolution kernels are 3 × 3 in size. In Figure 2, the first convolutional layer includes 32-channels convolution kernels for extract features of the input current signals, and the output result is input to the second convolutional layer and filtered with 64-channels convolution kernels. The third convolution layer connects the output of the second hidden layer with 128-channels convolution kernels. Then, we reshape the output size of the third hidden layer [4,900,512] to [1, 4 × 900 × 512], the fully connected layer takes the reshaped vector as input for feature integration. Finally, we predict the signal category through a softmax layer.

#### 2.4.3. Convolution Neural Network Using Abnormal Detection (CNN-AD)

CNN-AD is not a new type of neural network, but a method for solving the abnormal detection problem. Detection of tool breakage is a typical abnormal detection problem. In the solution of abnormal detection, the abnormal points often have a big difference from the normal points. So, models are trained only with positive samples and set a threshold to the classification that is often used in anomaly detection problems. In our experiments, tool breakage samples have a slight difference from the normal samples. The reason is that when the tool breakage occurs, the friction between the tool and the workpiece decreases instantly, which is reflected in a sudden drop in the current. The magnitude of the drop is affected by the tool material and the machine tool. Then, due to the compensation mechanism of the CNC [22], the friction will be compensated for quickly, and the current will recover and increase again. Finally, the goal of CNN-AD is to detect this anomaly on the signal.

Based on the advantages of CNN described in Section 2.4.1, we also expect that the model used in anomaly detection can help us find the difference between abnormal and normal samples for classification. Traditional machine learning, such as SVM, continuously improves the decision-making ability through the learning of samples, while CNN optimizes the feature learning of samples to find specific characteristics of the category to distinguish categories. CNN is more in line with our ideas. In addition, compared with other models, CNN has better fitting ability to high-dimensional data. Therefore, CNN is selected as the skeleton of anomaly detection in this work.

**Figure 3:**
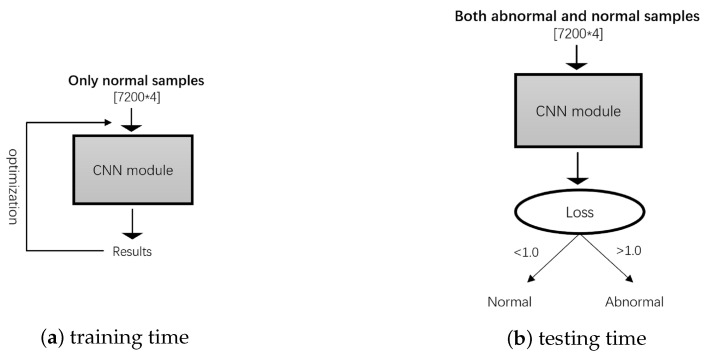
The architecture of CNN-AD. (**a**) In the training stage, CNN-AD is trained by only normal samples, the label is [1,0], then the cross entropy is calculated through the label and the output of the network, and Adam is used for network optimization. (**b**) In the test stage, CNN-AD divides the test set containing both normal and abnormal samples into two categories by threshold.

We use a five-layer CNN as the same as Section 3.5, train this model only with positive samples by gradient descent, and set a threshold according to the loss value. If the loss value of a sample is higher than the threshold, the model classifies it as a negative sample, as shown in Figure 3. It is worth noting that the threshold is not used in training, but only used to divide positive and negative samples in the test phase. We assume that the loss value of positive samples in the network is very small. But the negative samples will get a bigger loss value after passing through the network in the test stage, because the network has not seen the negative samples in the training stage. The initial setting of the threshold is manually set according to the loss value of the positive sample during training, which needs to be greater than the maximum loss value of the positive sample, and then the threshold is adjusted to be as small as possible to be less than the minimum loss value of the negative sample in the test stage to fully divide negative samples. For example, the maximum loss value in the training process is 0.5, setting the threshold to 0.6 will be an initial consideration, and then adjust the threshold according to the division effect in the test.

## 3. Results and Discussion

In this section, we try to find the current features that can reflect tool wear by the methods mentioned in Section 2.2. Then, these features we found will be fed into the models for training and test the model on the target dataset.

### 3.1. Time Domain Analysis

The processing log (Table 3) shows the life cycle of the tool and the processing volume of the machine. In the actual production, the CNC machine will be running until the tool has broken or the machine tool is abnormal. In this process, the worker is based on the experience of the machine processing noise to determine whether the tool edge is broken. When there is a sharp noise in the CNC machine, the worker will stop the machine and take out the tool and observe the breakage under a high-precision electron microscope. In fact, the worker’s judgment of the tool breakage will often be later than the actual moment.

**Figure 4:**
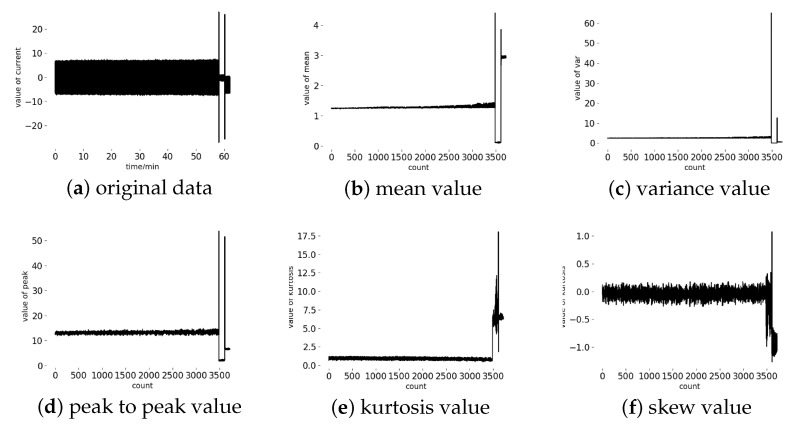
(**a**) the original data of a tool’s whole life cycle, (**b**) mean feature with a time span of 1 s, (**c**) variance feature with a time span of 1 s, (**d**) peak feature with a time span of 1 s, (**e**) kurtosis feature with a time span of 1 s, (**f**) skew feature with a time span of 1 s.

After wiping off the unnecessary current data, such as a reset signal, we analyzed the mean, variance, peak-to-peak, kurtosis and skew with a full life cycle of a tool. Figure 4a shows the whole life cycle of a tool. The rest of Figure 4, we compared the result of the time domain features with the original data. These features with a time span of 1 s have no obvious trend in the Figure 4, but we can observe that a mutation appeared in both of these features. Comparing Figure 4 and Table 3, it can be observed that the dramatic change of the current signal appears at 52–61 min and corresponds to abnormal in Table 3.

**Figure 5:**
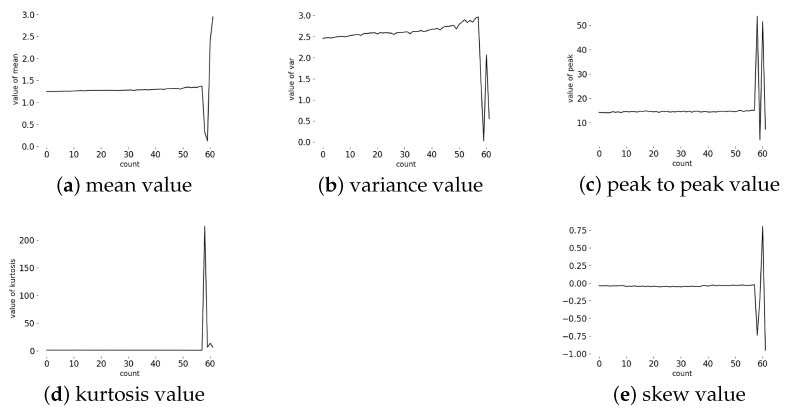
Time domain analysis with time span of one minute.

Figure 5 shows the analysis of the time domain with a time span of 1 min. Compared with Figure 4, the one-minute time domain features are more smooth and the upward trend is more obvious. It is easy to understand that increased time span is like smoothing the current signal. Through the experiments of different tools, we can conclude that the tool in this processing environment is windless in the earlier stage of life and it explains why the wear of the tool is smooth at the beginning and why a dramatic change appears at the end of its life.

### 3.2. Frequency Domain Analysis

**Figure 6:**
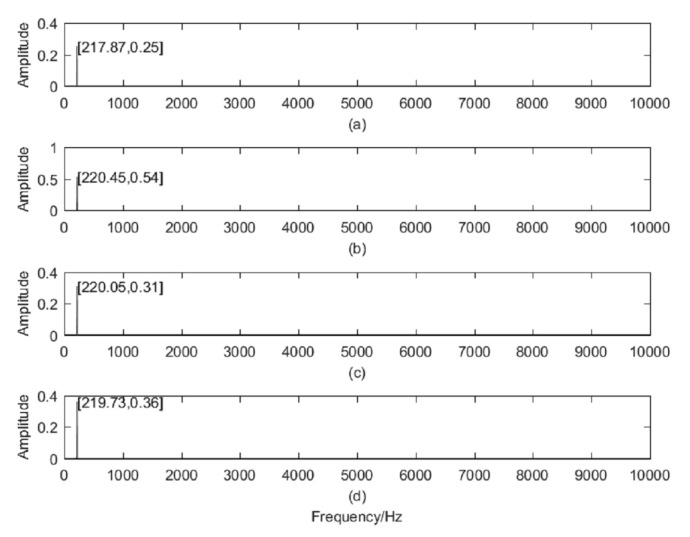
(**a**) FFT of new tool wear, (**b**) FFT of severe wear, (**c**) FFT of tool breakage, (**d**) FFT after tool breakage.

In this paper, Fast Fourier Transform (FFT) is used for data processing. The sampling rate of the data is 20,000 Hz, and the sampling point is 1,200,000/min. In order to obtain the change of the instantaneous frequency structure of tool breakage, we analyze the data of the initial processing, severe wear, tool breakage and after tool breakage. Figure 6a described the FFT of new tool wear, Figure 6b indicated the FFT of severe wear, Figure 6c responded to the FFT of tool breakage and Figure 6d is the FFT analysis after tool breakage. Concluding from Figure 6:

- The process frequency of the tool is mainly concentrated in the low frequency (220 Hz). And there are almost no amplitude of other frequency.
- The amplitude of cutting frequency varies with the change of tool state.

### 3.3. Wavelet Packet Analysis

**Figure 7:**
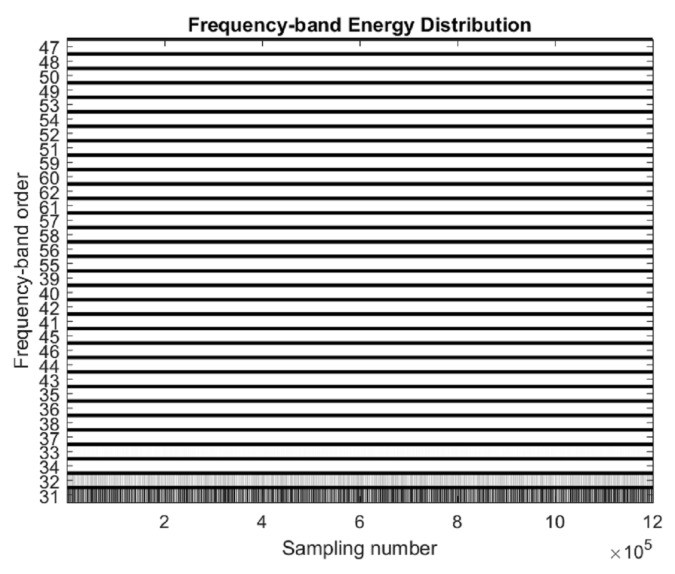
Frequency-band energy distribution at the initial stage of processing.

According to the results of frequency domain analysis, the wavelet packet decomposition level is 5, the corresponding frequency band resolution is 312.5 Hz, and the db5 wavelet is used for decomposition. The corresponding frequency-band energy distribution is obtained by analyzing the initial data (Figure 7).

In Figure 7, ordinates represent different frequency bands, with frequencies increasing from bottom to top. Each band has a bandwidth of 312.5 Hz, such as 31 for 0–312.5 Hz, 32 for 312.5–625 Hz, and 36 for the seventh frequency band of 1875–2187.5. The abscissa indicates the time. The darker the color of the frequency band is, the higher the energy is.

It can be seen from Figure 7 that the energy of the signal is mainly distributed in the range of 0–624 Hz, and the frequency band of 0–312.5 Hz has more energy, which is consistent with the fourier analysis results. The energy distribution of the signal is determined, and then, the energy change of the two cycles is analyzed.

**Figure 8:**
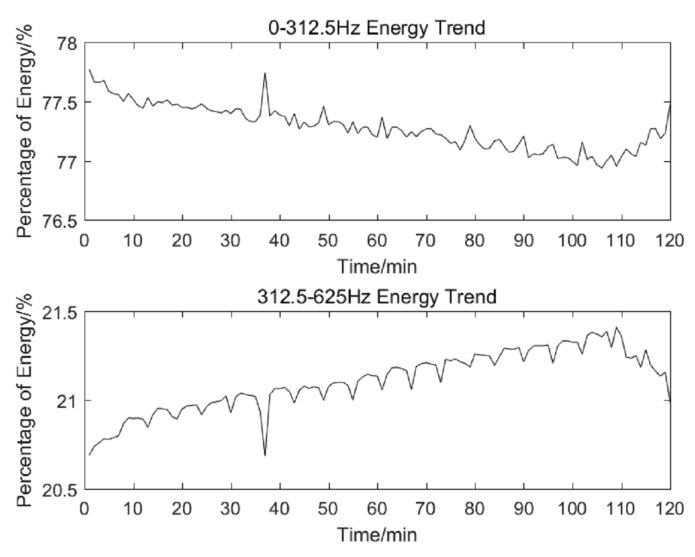
Energy trend of the top two frequency bands, the top picture described the energy change of the band 0–312.5 Hz, the bottom picture indicated the energy change of the band 312.5–625 Hz.

As shown in Figure 8, the trend of the two frequency bands is the opposite, the frequency band of 0–312.5 Hz (recorded as frequency band 31) energy: 1. With the increasing processing time, energy gradually decreases. 2. After tool breakage, the energy gradually increases. Besides, the energy of band 312.5–625 Hz (recorded as band 32) increases with the increasing of processing time and decreases after tool breakage. At the same time, the extreme points of Figure 8 in 37 min is caused by the machine tool stopped processing.

### 3.4. Data Processing and Feature Selection

In the experiment, before the data enters the model training, denoising processing and feature extraction are required to improve the correlation between the data and tool wear. The signals are mainly composed of three parts—useless machine idling signals, processing signals and noise signals. In this section, we expect to clarify the frequency of each part through the frequency technology, so as to filter out the noise signal, making the data cleaner.

**Figure 9:**
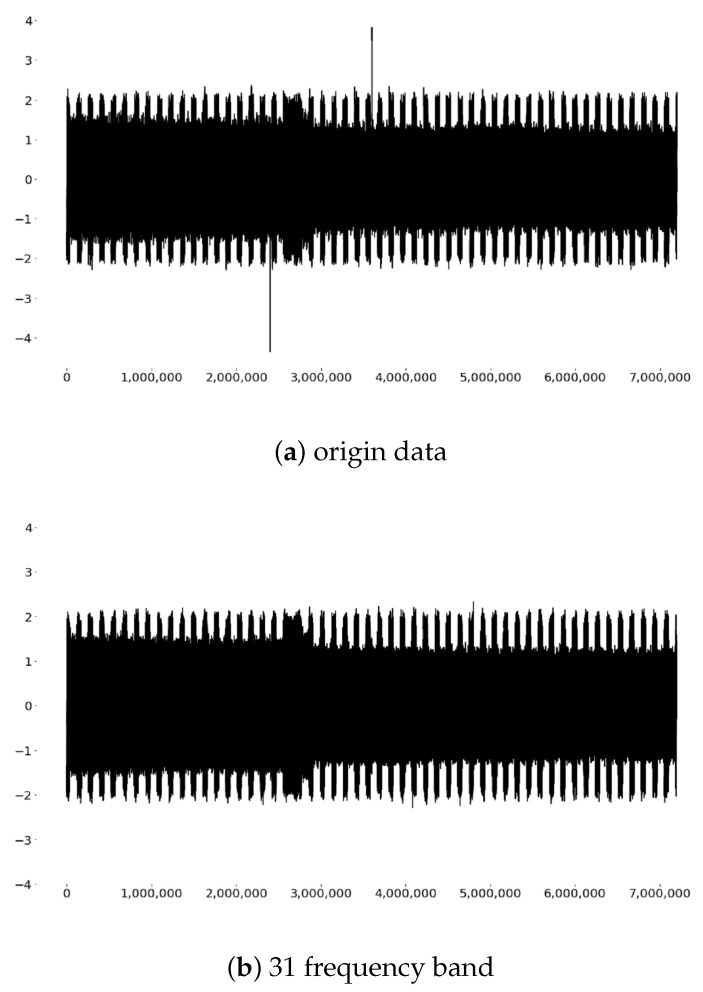

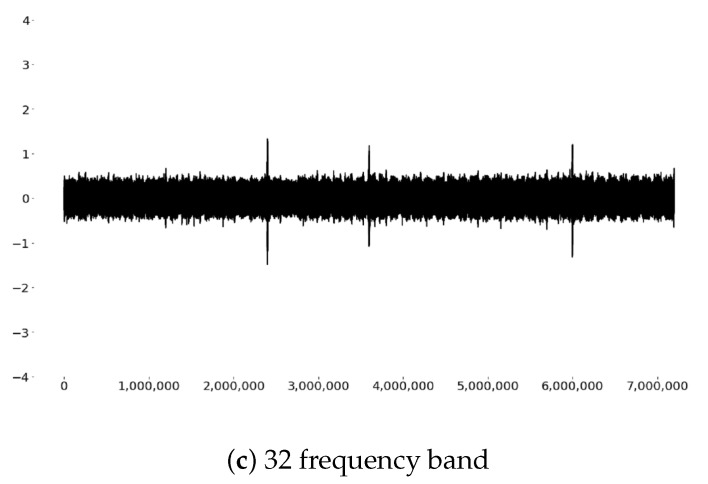
(**a**) The original data of a current sample. (**b**) The original data is filtered by 300 Hz low-pass. It can be seen that the two high amplitudes in the original data are filtered out, and the filtered signal is smoother. (**c**) The original data is filtered by the frequency band of 300 Hz to 600 Hz, and it can be seen that the signal amplitude after filtering is small and disorderly. We regard it as current noise.

According to the results of the Fourier analysis in Section 3.2, the signal is mainly composed of 220 Hz signals, but Fourier cannot reflect the changes in signal energy in the time dimension. Wavelet packet technology solves this problem well. The result of wavelet analysis shows that the signal has energy in both the 31 and 32 bands, and the 31 band has the strongest energy, which is consistent with the Fourier results. There is a certain correlation between the tool wear and the energy trends of all two frequency bands. Therefore, we used low-pass and frequency band filtering to figure out the components of these two frequency bands, as shown in Figure 9.

It can be concluded that the signals in the 31 frequency band is the tool processing signal which is more flat, while the 32 frequency band seems to be composed of processing noise. Therefore, we use a low-pass filter to denoise the signals, and then the denoised signals after feature extraction are input into the model for training.

**Figure 10:**
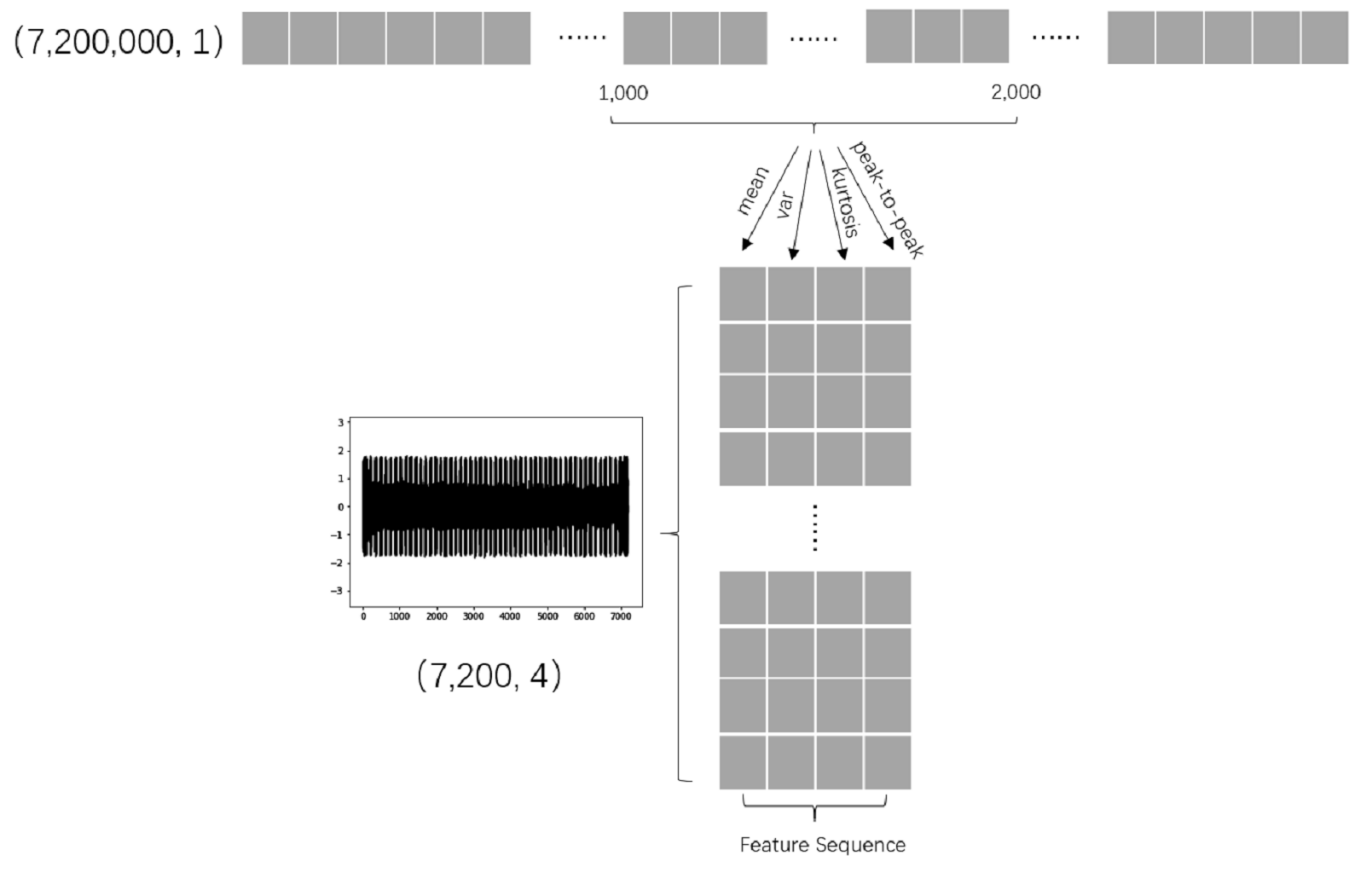
The characteristic calculation is carried out at every 1,000 points, so that the original current data of 6 min is under-sampled to 7200 × 4 (four characteristics).

After signal filtering, we use time domain technology to extract features of the signals (described in Section 3.1). Considering that the skewness does not have a good effect on all cutting tools, it reflects the degree of symmetry of the signal, while the current signal performs better in symmetry in the experiment. We choose the four characteristics of mean, variance, kurtosis, peak-to-peak value as the input of neural network, indicating the stability of the signal, the degree of fluctuation, the amplitude of the fluctuation, and the abnormal situation, in order to comprehensively characterize the change information of tool wear on the current signal. Finally, we get the input data of the network by under-sampling with the four feature methods, as shown in Figure 10.

### 3.5. Tool Breakage Detection by Models

In the experiment, we regard the prediction of tool breakage as two categories game, normal samples are taken as positive samples, and vice versa. The structure of CNN-AD is shown as Section 2.3, four characteristics are taken as input for training. In order to verify the effect of CNN-AD, we take three commonly used models as a comparison, CNN model without anomaly detection, back propagation network (BP) and SVM. The both structure and input of CNN model without anomaly detection is the same as CNN-AD, except the way of training. BP network consists of a three-layer perceptron with 512 neurons. The dimension of input data was reduced from 7200 × 4 to 16 by principal component analysis (PCA), and then taken into SVM. In addition, we added an unsupervised anomaly detection method for comparison, One-Class Support Vector Machine (OCSVM) which is introduced by Schölkopf et al. [23] OCSVM is about to learn a rough, close frontier delimiting the contour of the initial observations distribution, then, if further observations lay within the frontier-delimited subspace, they are considered as coming from the same population than the initial observations. Otherwise, if they lay outside the frontier, they will be judged abnormal. In the experiment, we chose the RBF kernel for OCSVM to define the boundary.

In the training stage, both positive and negative samples are used for training in the comparison models, and for CNN-AD, there are only positive samples. In the testing stage, CNN-AD predicts the sample as normal or abnormal by a threshold (set empirically to 1.0) to divide the network loss. Besides, due to the class-imbalance of samples, the accuracy can not correctly evaluate the performance of models on the abnormal samples, we use confusion matrix, recall rate and F1-score as reference index to evaluate the performance on both normal and abnormal samples. The average results of these models reported in Table 4. In the experiment, we chose the RBF kernel for OCSVM to define the boundary.

We can conclude from Table 4 that CNN-AD performs best, the accuracy rate is 100%, which is possibly caused by the training way of CNN-AD can amplify the difference between normal and abnormal samples. CNN-AD did not learn the characteristics of abnormal samples which results in the high loss value of abnormal samples.

**Figure 11:**
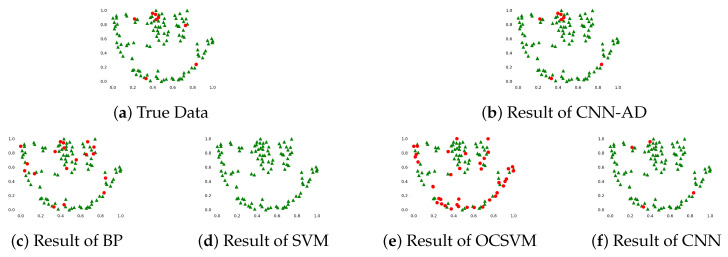
The prediction results of each model in the testing stage. The test data is reduced to 2 dimensions by t-sne, red indicates abnormal samples, green indicates normal samples.

The difference between positive samples was small, from the confusion matrix of each model (Table 5), the performance of models in positive samples is satisfactory, SVM, CNN and CNN-AD can even achieve 100% accuracy of positive samples. The main difference in the performance of these models lies in the prediction of negative samples, the prediction accuracy of each models in negative samples was 75% (BP), 0% (SVM), 12.50% (OCSVM), 50% (CNN), 87.5% (CNN-AD) respectively. In the comparison of anomaly detection methods, OCSVM is better than SVM in judging abnormal samples, while the judging performance of normal samples is poor. This may be due to the small difference of the sample points in the multi-dimensional space (Figure 11), and the sample points cannot be divided directly by the boundary.

The difficulty of predicting tool breakage lies in the prediction accuracy of negative samples, as shown in Figure 11, the abnormal samples are mixed between the normal samples, and the difference between the abnormal and the normal samples cannot be clearly observed from the two-dimensional representation of the current data. In addition, the time of tool breakage is very short compared to normal processing, there are only a few abnormal samples but dozens of positive samples of a whole life cycle of a tool. The form of each breakage tool in current signals is likely to be different, such as the difference in current amplitude or the changing trend. These reasons make the model can not fit the negative sample very well. CNN-AD solves this problem by enlarging the difference between positive and negative samples by its unique training method.

**Figure 12:**
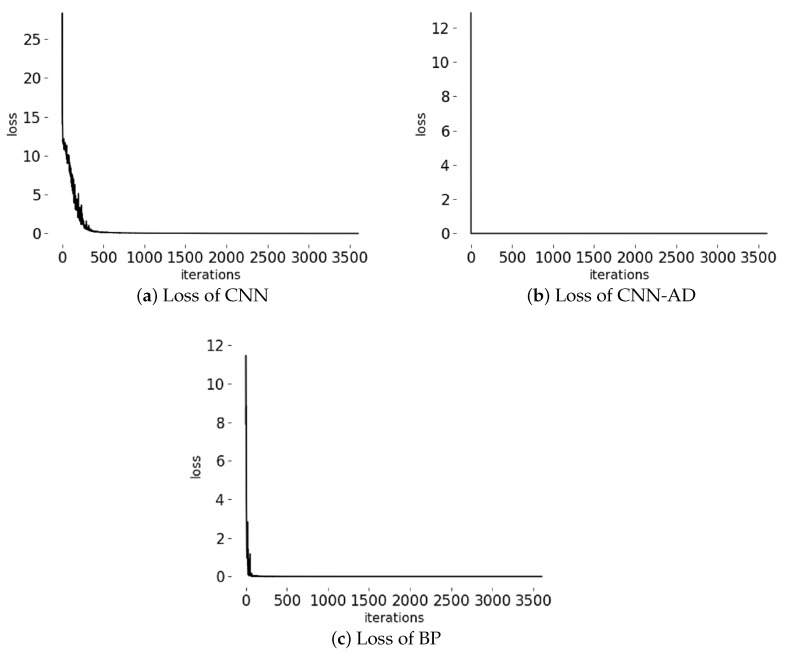
Loss change in model iteration.

Figure 12 shows the loss of CNN-AD, CNN and BP; it indicates that normal process samples are similar and easier to fit. During the training step, CNN-AD reaches convergence just in 10 iterations, that is because CNN-AD is trained only with positive samples, and positive samples are very similar to each other, which is due to fast convergence. CNN convergence requires 300 iterations, BP reaches convergence in 100 iterations. Although BP converges faster than CNN, the results of BP are fluctuating. BP is not stable, its accuracy often has more than 80% or less than 60%. CNN is stable, the accuracy of CNN is stable at more than 95%. This may be because CNN is more complex and has more parameters than BP.

**Figure 13:**
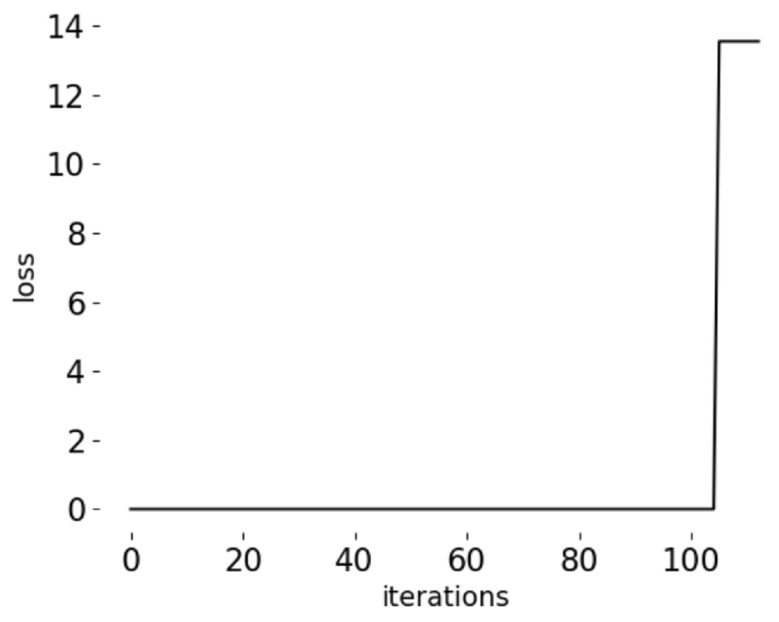
Loss of CNN-AD in test stage, the sudden rise of loss value is the moment of tool breakage.

Figure 13 shows the loss of the CNN-AD in the test stage, test dataset is a continuous life cycle of a tool, we can observe that the loss of the network suddenly increases in the late life of the tool, which is consistent with our previous analysis. The characteristics of the tool breakage are very different from the characteristics of the normal sample, so we can classify abnormal samples by the threshold.

## 4. Conclusions

In this paper, we find out some characteristics related to tool wear by feature engineering. Then, we propose a simple and effective CNN-based anomaly detection method to detect tool breakage through the spindle current, and verify the feasibility that tool breakage of CNC can be detected by spindle current. Finally, we give evidence that the anomaly detection method is more suitable for the problem of positive and negative sample imbalance. There is still some problem of limitations of the method, the performance of CNN-AD is based on the difference in the distribution between the abnormal and the normal samples. In addition, CNN-AD only learns from normal samples, it does not have the ability to learn abnormal samples. When the data distribution of the abnormal samples is very close to the normal samples, the performance of CNN-AD may decrease. In addition, because the negative data in the experiment are not sufficient to cover all forms of tool breakage, it can not verify the performance changes of CNN-AD on the above problems. Different machine tools and different tools have a different form of tool breakage, our results in this paper are built for a given machine with specific parameters, tools and materials. If this environment is changed, the results will be different, because the wear of tools is affected to a large extent by this environment element. So, if you plan to use CNN-AD, please make sure there is a difference between positive and negative samples.

In view of the limitations of CNN-AD, we plan to introduce the transfer learning [24] to solve these problem, the processing data under different parameters but similar distribution is transferred for the tool wear prediction under the target parameters to cover more abnormal situations and make the model have the learning ability of normal samples and abnormal samples for supervised learning. We expect this approach can bring better generalization capabilities and be more suitable for actual scenarios.

## Figures and Tables

**Table 1:** Experimental environment.

Parameters	Values
Machine model	Super MC F2.0-I/S
Tool type	Corner Radius End Mill
Tool material	Cemented carbide
Tool specification	701B-DJ 4244-50
Workpiece material	S136
Processing method	Milling
Rotating speed	6500 r/min
Feed rate	1500 mm/min
Working Engagement	2.0
Back Engagement	0.1

**Table 2:** Some parameters of neural network.

Parameters	CNN
Network depth	5
Learning rate	0.0001
Activation function	Relu
Size of input	7200 × 4
Mini-batch	20
Total number of cycles	3600
Optimizer	Adam

**Table 3:** The processing log.

Start Time	End Time	Cutting Sound Size
0	17	slight
12	40	normal
40	52	slightly larger
52	61	abnormal

**Table 4:** Performance of models.

Model	Accuracy	Recall Rate	F1-Score
BP	87.6%	88.57%	88.08%
SVM	92.92%	100%	91.892%
OCSVM	60.18%	63.81%	61.94%
CNN	96.46%	100%	97.87%
CNN-AD	99.12%	100%	99.55%

**Table 5:** Confusion Matrix of Models.

Confusion Matrix			The Actual Data	
			Positive	Negative
BP	Predict data	positive	93	2
		negative	12	6
SVM	Predict data	positive	105	8
		negative	0	0
OCSVM	Predict data	positive	67	7
		negative	38	1
CNN	Predict data	positive	105	4
		negative	0	4
CNN-AD	Predict data	positive	105	1
		negative	0	7
